# Systemic lupus erythematosus presenting as Evans syndrome

**DOI:** 10.1002/ccr3.2550

**Published:** 2019-11-18

**Authors:** Liya Stolyar, Bijan Rizi, Sonia Lin, Eric Hsieh

**Affiliations:** ^1^ Department of Internal Medicine University of Southern California Los Angeles California

**Keywords:** SLE Evans thrombocytopenia

## Abstract

Hematologic abnormalities are an important part of the diagnostic criteria for systemic lupus erythematosus (SLE). This case presents a patient diagnosed with Evans Syndrome with underlying SLE on initial presentation.

## Graphical abstract

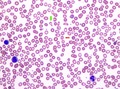

## 1 CASE PRESENTATION

A 33‐year‐old male patient with no significant medical history presented with generalized weakness, fatigue, lightheadedness, and easy bruising for one month. His symptoms gradually worsened over the course of the month to where he would stand up and immediately feel dizzy. He also reported bruising on his lower extremities and blood‐filled oral blisters that would intermittently burst and heal spontaneously. Examination was significant for hyperpigmented lesions on the forehead; multiple ulcerating and blistering oral lesions with dried blood on the tongue, gums, and posterior pharynx; multiple ulcerations and necrotic oral lesions on the left retromolar trigone, tongue, and hard palate; bilateral lower extremity petechiae; and dark, nonpalpable, and necrotic lesions on the shins.

**Figure 1:**
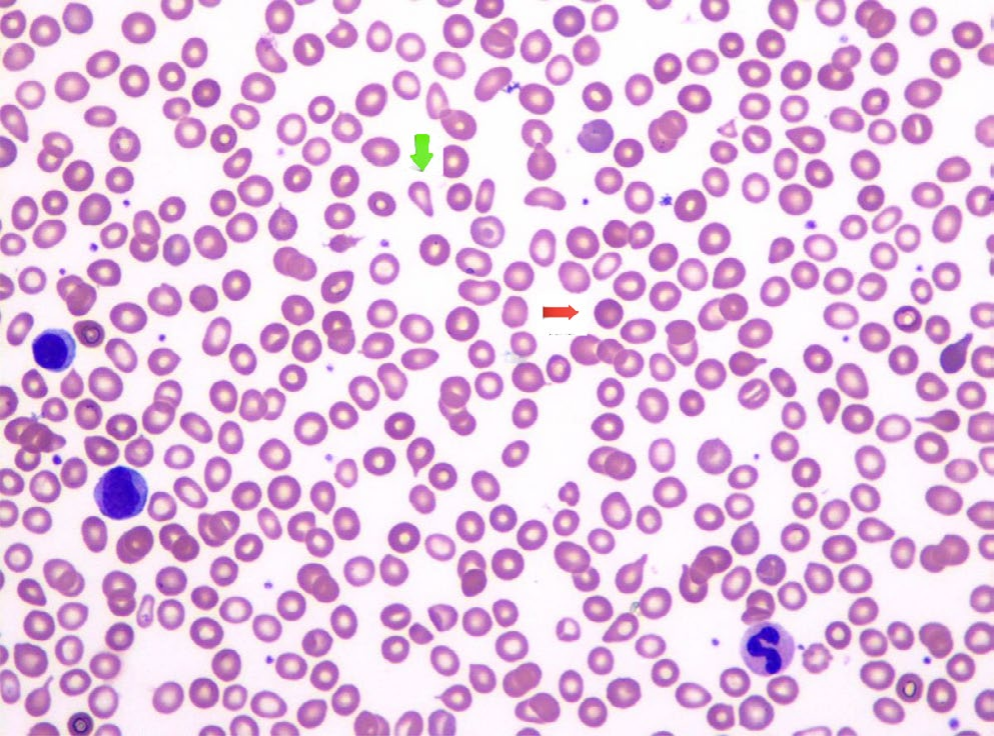
Anisopoikilocytosis (variance in size and shape) is moderately increased by tear drop cells (green arrow), spherocytes (red arrow), microcytes, and nonspecific poikilocytes. The anisopoikilocytosis is concordant with the finding of a mild increase in marrow reticulin fibrosis. Hematopathology slide prepared at LAC + USC Medical Center by Dr Wendy Kadi, DO and Dr Maria Vergara‐Lluri, MD

Laboratory testing revealed severe normocytic anemia (Hb 48 g/L, MCV 92.6 fL) and thrombocytopenia (platelets 6 × 10^3^/L). Laboratories were also notable for haptoglobin <1 µmol/L, LDH 14.3333 µkat/L, D‐dimer 2269 2269 μg FEU/L, positive direct Coombs test, and a peripheral smear demonstrating fragmented red blood cells (see Figure 1). Urine analysis showed urine protein/creatinine ratio of 0.31. Further laboratories showed low C3 (0.78 g/L) low C4 (0.27 µmol/L), serum IgG 234.96 µmol/L, ANA 1:80 homogenous, positive beta‐2 glycoprotein IgM, a detectable lupus anticoagulant, and positive anticardiolipin IgM. A biopsy of the oral ulcer was done, which did not show immune complexes.

Given his constellation of clinical and laboratory findings, the patient was diagnosed with Evans Syndrome related to SLE, as evidenced by his hemolytic anemia, positive direct Coombs test, and immune thrombocytopenia. The patient received pulse IV methylprednisolone for 3 days and was then transitioned to daily prednisone. He also received two doses of IVIG and one dose of romiplostim for treatment of his Evans Syndrome. For management of his SLE, he was initiated on weekly rituximab. He had subsequent symptomatic improvement, with resolved oral lesions, as well as continued improvement in his anemia and thrombocytopenia. At the last follow‐up, he continued to improve and remained stable on mycophenolate mofetil.

## 2 DISCUSSION

This case underscores the importance of considering Evans Syndrome in patients with underlying rheumatologic conditions such as SLE.^1^, ^2^, ^3^ Patients with hematologic manifestations of SLE, such as thrombocytopenia and autoimmune hemolytic anemia, should be considered for Evan Syndrome.^1^, ^2^, ^3^ As Evan syndrome is a diagnosis of exclusion, a differential diagnosis of Evan syndrome is key and includes the following: autoimmune lymphoproliferative syndrome, paroxysmal nocturnal hemoglobinuria, thrombotic thrombocytopenic purpura, hemolytic‐uremic syndrome, antiphospholipid syndrome, Sjogren syndrome, IgA deficiency, lymphomas, chronic lymphocytic leukemia, and AIHA caused by drugs.^4^ An accurate diagnosis of Evan syndrome is important as the management may differ from a patient who is diagnosed with SLE alone.^1^, ^2^, ^5^, ^6^ Whereas cases of SLE alone would be treated with prednisone and rituximab, management of SLE with Evan syndrome should be supplemented with romiplostim.^7^ Proper and rapid identification of this case of Evan Syndrome led to appropriate therapy with immunosuppressive agents that allowed him to recover more quickly and regain hematologic function.^1^, ^2^, ^3^, ^5^

## CONFLICT OF INTEREST

None declared.

## AUTHOR CONTRIBUTIONS

Liya Stolyar MD: drafted initial document and performed acquisition, analysis, and interpretation of data. Bijan Rizi: made significant contributions to conception and design of the manuscript and performed interpretation of data. Sonia Lin MD: edited manuscript and revised aspects important for intellectual content and gave final approval for manuscript to be published. Eric Hsieh MD: edited manuscript and revised aspects important for intellectual content and gave final approval for manuscript to be published.
